# Supplementary material for: Cesium-mediated electron redistribution and electron-electron interaction in high-pressure metallic CsPbI3
Source: Nat Commun. 2022 Nov 18;13:7067. doi: 10.1038/s41467-022-34786-5 (PMC9674642; doi:10.1038/s41467-022-34786-5)
Supplement: Supplementary file 1 — Supplementary Information [file 41467_2022_34786_MOESM1_ESM.pdf]

Supplementary information for

Cesium-mediated electron redistribution and electron-electron interaction in high-pressure metallic $CsPbI_3$

Feng Ke, Jiejuan Yan, Shanyuan Niu, Jiajia Wen, Ketao Yin*, Hong Yang, Nathan R. Wolf, Yan-Kai Tzeng, Hemamala I. Karunadasa, Young S. Lee, Wendy L. Mao, Yu Lin*

*To whom correspondence should be addressed. E-mail: yinketao@lyu.edu.cn; lyforest@stanford.edu

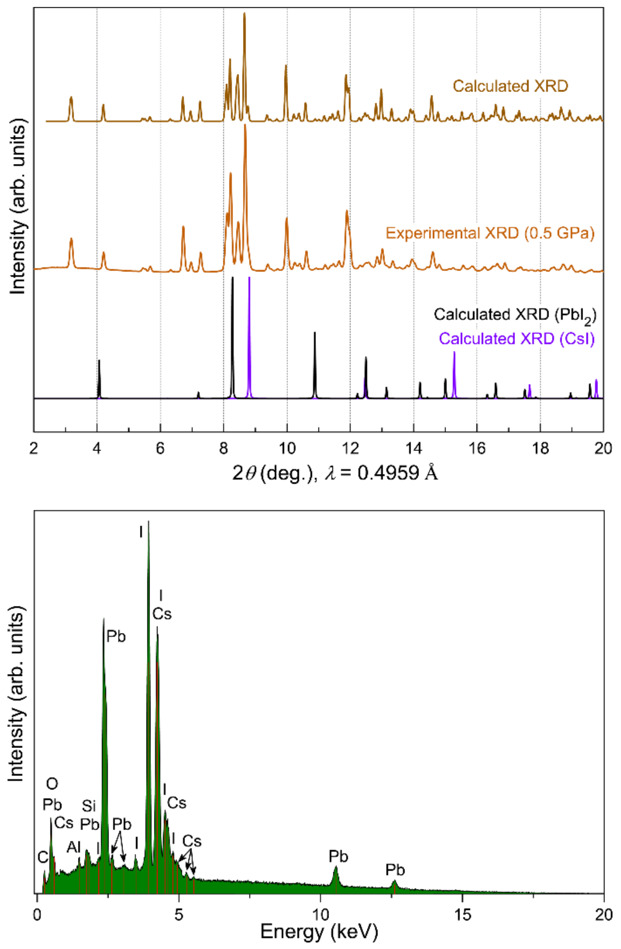

Supplementary Fig. 1. Structural and compositional check of the synthesized δ-$CsPbI_3$ starting samples. Top panel, XRD result for the phase purity. The XRD of the starting sample matches the simulated pattern of δ-$CsPbI_3$, and no peaks from other phases or the starting precursors (CsI and PbI_2) are observed, indicating the phase purity of δ-$CsPbI_3$. Bottom panel, energy-dispersive X-ray spectroscopy measurement for elemental analysis. No elemental impurity is in the sample. The Al peak comes from the holder, the Si peak from the wafer, and C and O from environmental background.

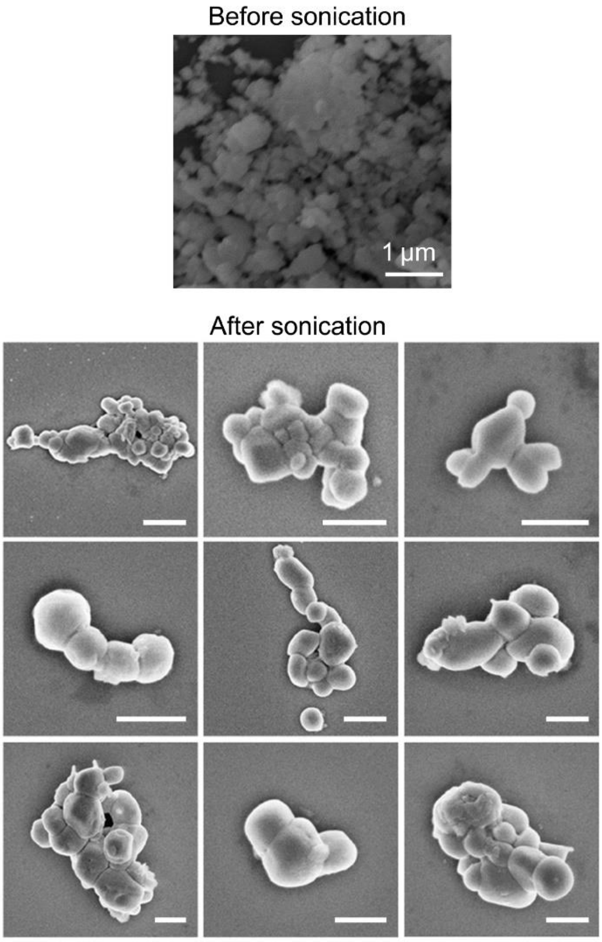

Supplementary Fig. 2. SEM images of the synthesized δ-$CsPbI_3$ sample used for the electrical transport and XRD measurements. Top panel, the SEM image of the powdered sample before sonication. Bottom nine panels, SEM images of the sample after one hour of sonication in hexane. Statistical analysis show that the particles are submicron-sized and are mainly between 100 – 400 nm. The scale bars in the bottom nine figures are 200 nm.

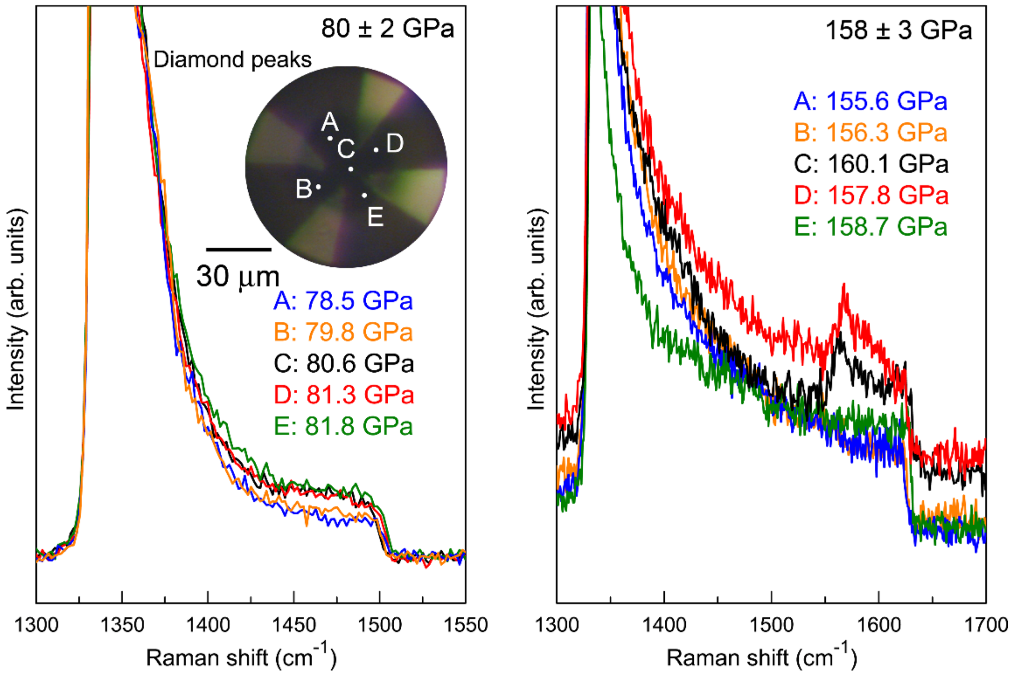

Supplementary Fig. 3. Pressure determination for the high-pressure electrical transport measurements using the Raman peak of diamond. Raman spectra at two representative pressures, i.e., ~80 GPa (left) and ~158 GPa (right), are shown from different locations in the sample (inset image). The pressures above 30 GPa were determined by taking the average values of the pressures measured at various positions at the center and the edge of the sample.

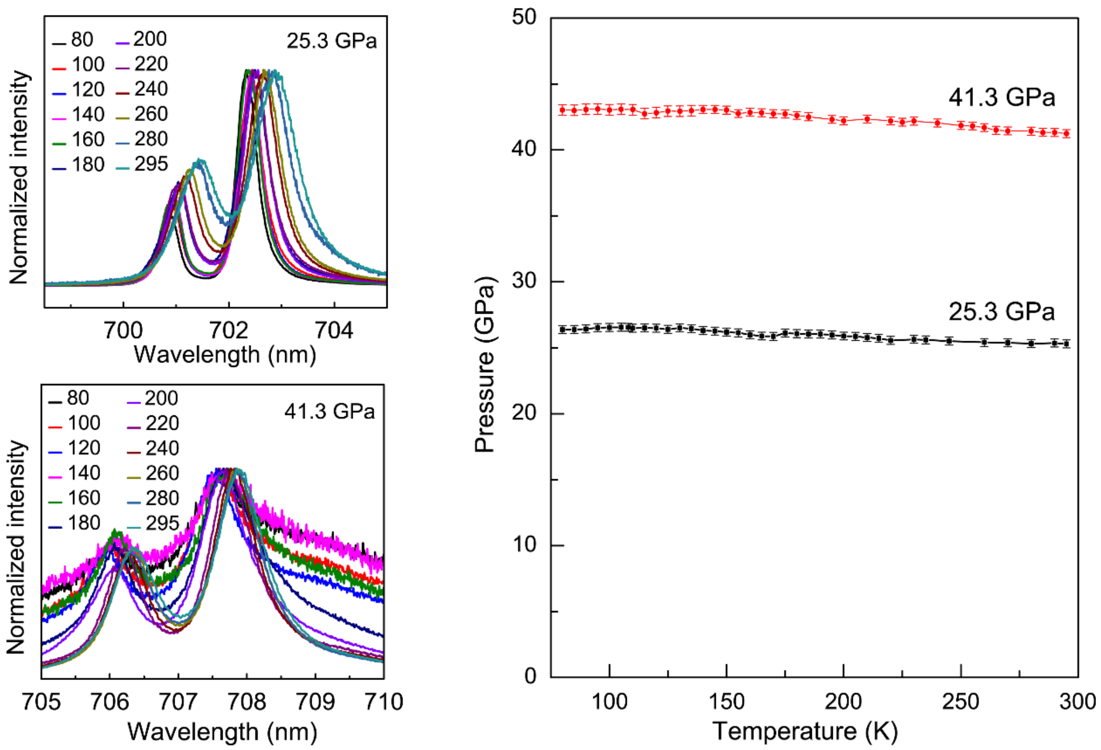

Supplementary Fig. 4. Pressure stability test of the Be-Cu cell used for the electrical transport measurements at low temperature. Left two panels, temperature dependence of the ruby fluorescence at high pressure. Right panel, the calculated pressures as a function of temperature. Our tests show that the pressure is stable and only has a slight drift (< 2 GPa up to 50 GPa) from room temperature down to 78 K. This agrees well with previously reported results based on a similar PPMS-DAC, which showed that the pressure shift was < 2 GPa down to 150 K followed by a smaller pressure shift of < 0.5 GPa with further cooling to 5 K at ~ 45 GPa[1]. Above 50 GPa, the ruby fluorescence is too weak to be detected at low temperature. The error bars are from the temperature uncertainty during the measurements.

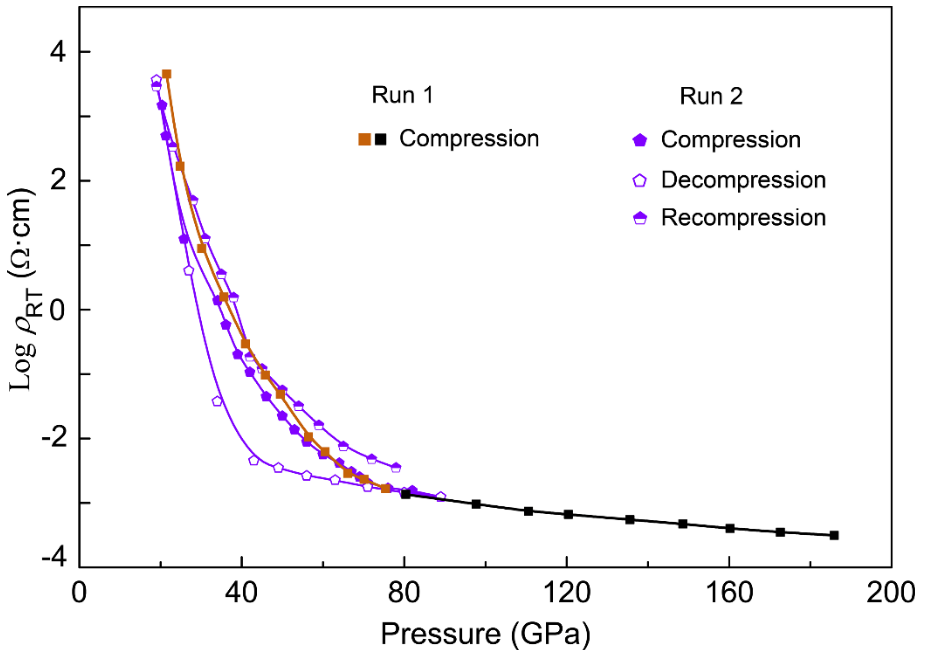

Supplementary Fig. 5. Room-temperature resistivity (Log ρ_{RT}) of $CsPbI_3$ at pressure cycles. In run 1, beveled 100-μm diamond anvils were used for the resistivity measurements up to 186 GPa. In run 2, beveled 150-μm diamond anvils were used for measurements over two pressure cycles. The sample was compressed up to 90 GPa, and then decompressed to 10 GPa, followed by recompression to 80 GPa.

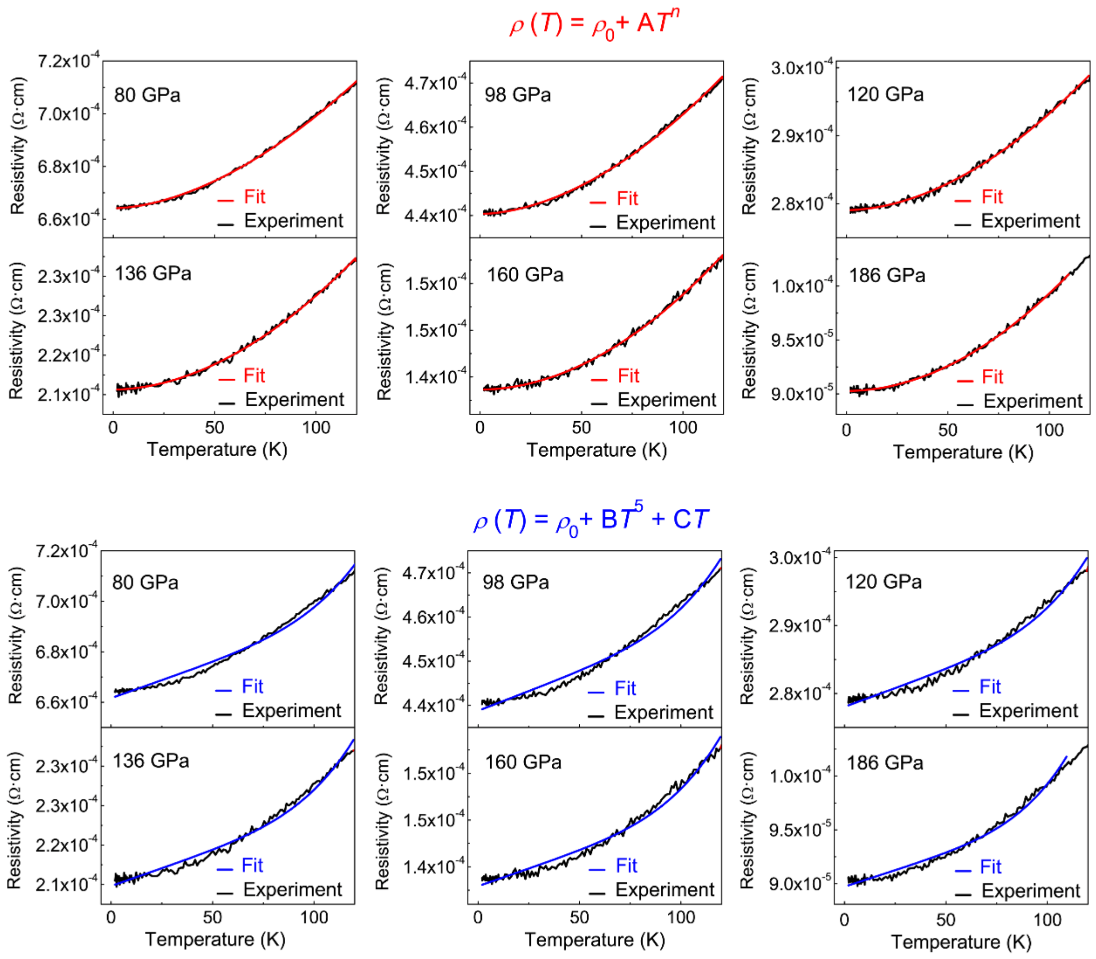

Supplementary Fig. 6. Fitting of the $\rho - T$ curves with different models. Top six panels, fitting results (red lines) using the $\rho(T) = \rho_0 + AT^n$ model. Bottom six panels, fitting results (blue lines) using the $\rho(T) = \rho_0 + BT^5 + CT$ model.

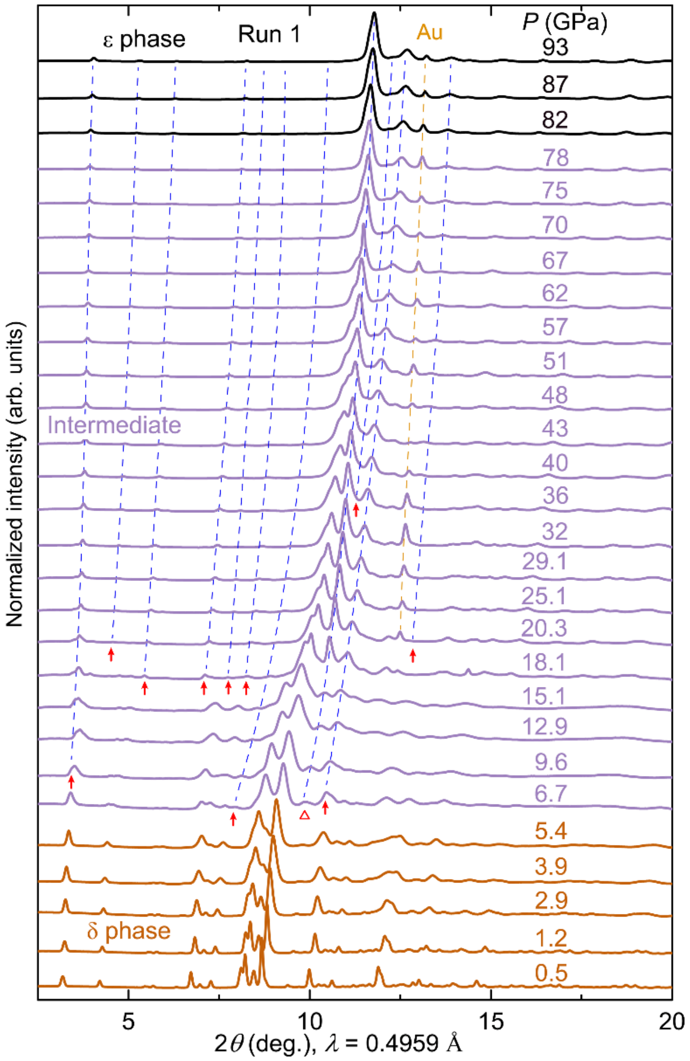

Supplementary Fig. 7. Full set of XRD patterns of $CsPbI_3$ as a function of pressure in run 1. 200-µm diamond anvils and neon pressure-transmitting medium were used. Pressure was calibrated using ruby and gold powders in the vicinity of the $CsPbI_3$ sample. The red arrows mark the appearance of new diffraction peaks. The red triangle marks the diffraction peak at 9.8° which rapidly increases in intensity and becomes the strongest peak above 20.3 GPa. The blue dashed lines indicate the evolution of the sample peak positions with pressure. The yellow dashed line tracks a diffraction peak of gold (Au) which is the internal pressure standard.

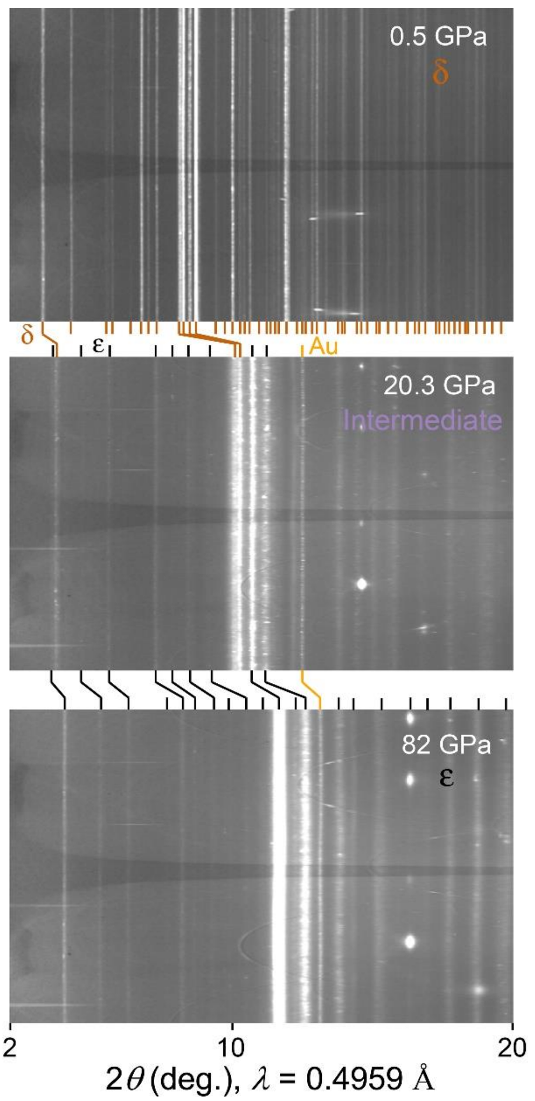

Supplementary Fig. 8. Caked XRD patterns of $CsPbI_3$ at 0.5, 20.3, and 82 GPa in run 1. The diffraction lines that correspond to δ-$CsPbI_3$, ε-$CsPbI_3$, and the internal pressure standard Au are marked as orange, black and yellow, respectively. The diffraction lines at large diffraction angles ($2\theta > 12$ degrees) in the 20.3 GPa pattern are not labeled because in the mixed phase regime, δ-$CsPbI_3$ and ε-$CsPbI_3$ alone or in combination contribute intensities to these already weak diffraction peaks, making peak assignment impossible.

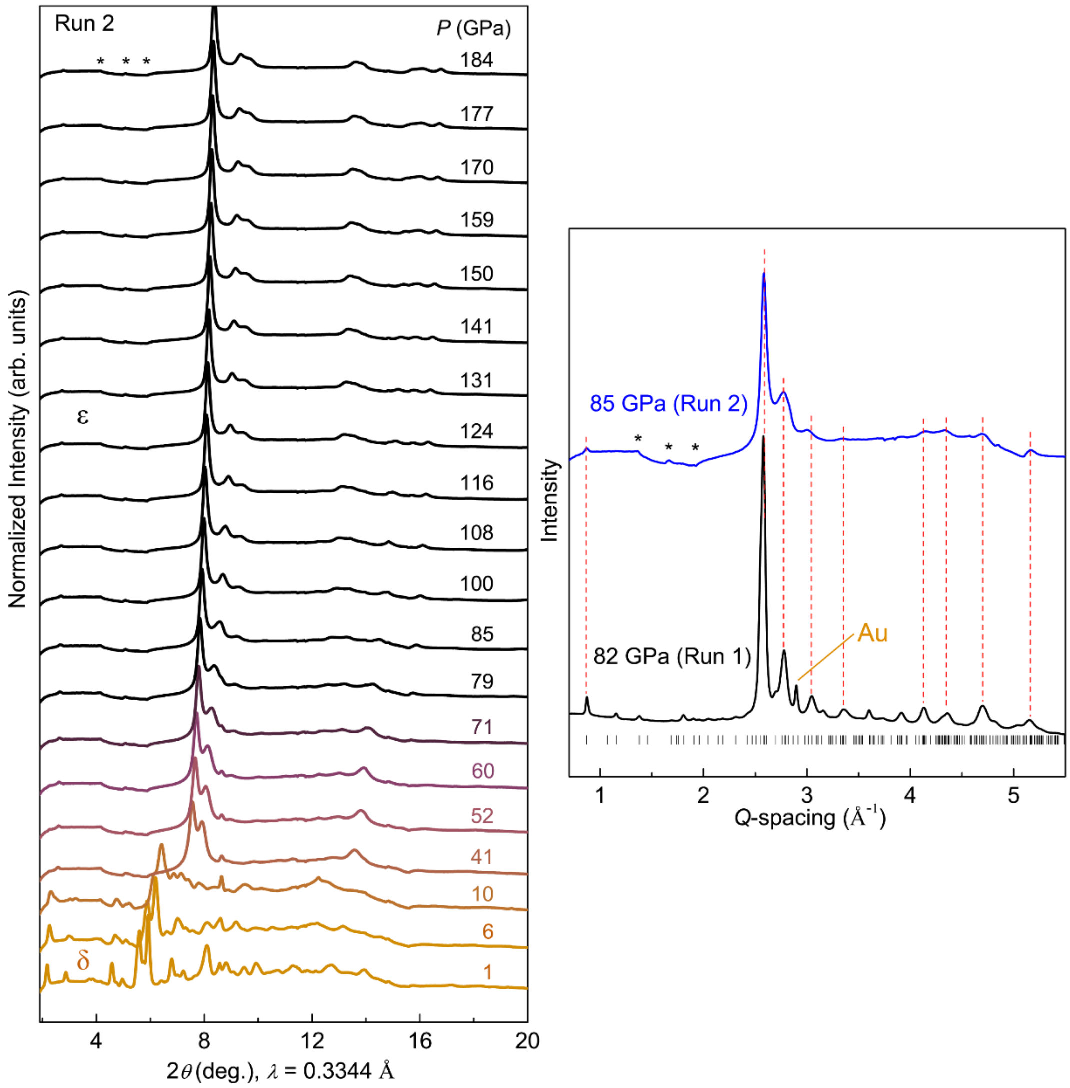

Supplementary Fig. 9. XRD results of $CsPbI_3$ in run 2. Left panel, XRD patterns of $CsPbI_3$ up to 184 GPa. The weak peaks marked by the black asterisks are background from the detector, which are pressure-invariant. Right panel, XRD patterns at 85 GPa in run 2 and 82 GPa in run 1 for comparison. The XRD patterns of the high-pressure ε phase in run 2 are in excellent agreement with those in run 1 (Supplementary Fig. 7), except for slight differences in the relative peak intensities and widths. To reach up to 180 GPa, 100-μm culet and a thin sample were used in run 2 that significantly reduces the sample diffraction volume, resulting in the overall weak intensities in the

diffraction patterns and some indistinguishable peaks. In addition, the small culet size makes the pressure control in the low-pressure regime difficult, especially with the gas membrane, so we were not able to collect as many diffraction patterns below 40 GPa. In run 2, a much shorter wavelength (λ = 0.3344 Å) was used versus λ = 0.4959 Å in run 1, which results in the XRD patterns having a relatively lower angular resolution compared to run 1. This leads to closely spaced peaks being merged in run 2, such as those peaks around the strongest reflection at ~8.0°. Although the larger pressure step at low pressures and the relatively lower angular resolution make it challenging to precisely determine the starting/ending pressures of the sluggish *Pnma*-to-$Pmn2_1$ structural transition from run 2, we are confident that the high-pressure ε phase persists up to 184 GPa.

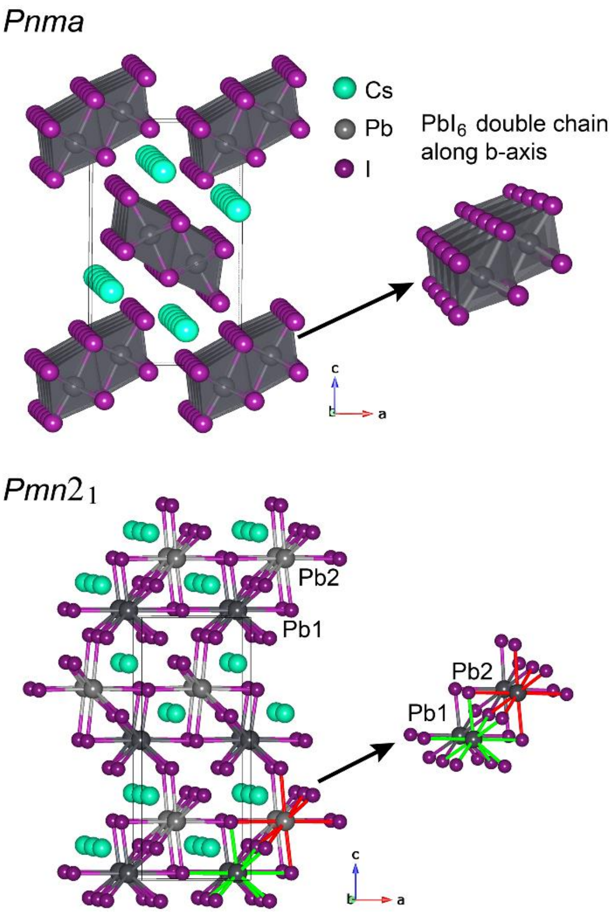

Supplementary Fig. 10. Structural models of $CsPbI_3$. Top panel, structural model of the *Pnma* (δ) phase showing the double chains of PbI_6 octahedra along the b-axis. Bottom panel, structural model of the high-pressure *Pmn*2_1 (ε) phase showing the nine- and eight-fold coordination of Pb1 (dark gray) and Pb2 (light gray) atoms with the I atoms, respectively.

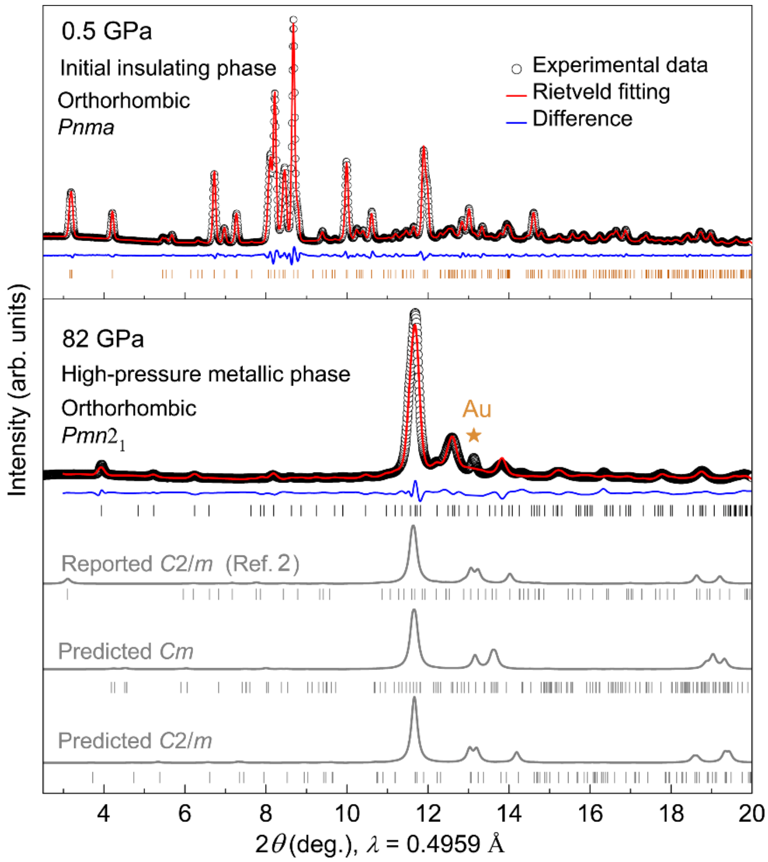

Supplementary Fig. 11. Rietveld refinement of the XRD patterns using the GSAS software. Top panel, refinement results of the XRD pattern at 0.5 GPa in run 1 with the *Pnma* structure (R_{wp} = 3.2%). The obtained lattice parameters are a = 10.437±0.004 Å, b = 4.789±0.002 Å, c = 17.716±0.006 Å. Bottom panel, refinement results of the XRD pattern at 82 GPa with the $Pmn2_1$ structure (R_{wp} = 7.6%). The obtained lattice parameters are a = 5.869±0.006 Å, b = 4.516±0.005 Å, c = 14.459±0.011 Å. The gray lines at the bottom are the simulated XRD patterns from a previously reported $C2/m$ structure[2], and the predicted Cm and $C2/m$ structures from our calculations at 82 GPa. Our XRD patterns above 82 GPa cannot be indexed into any of these three structures.

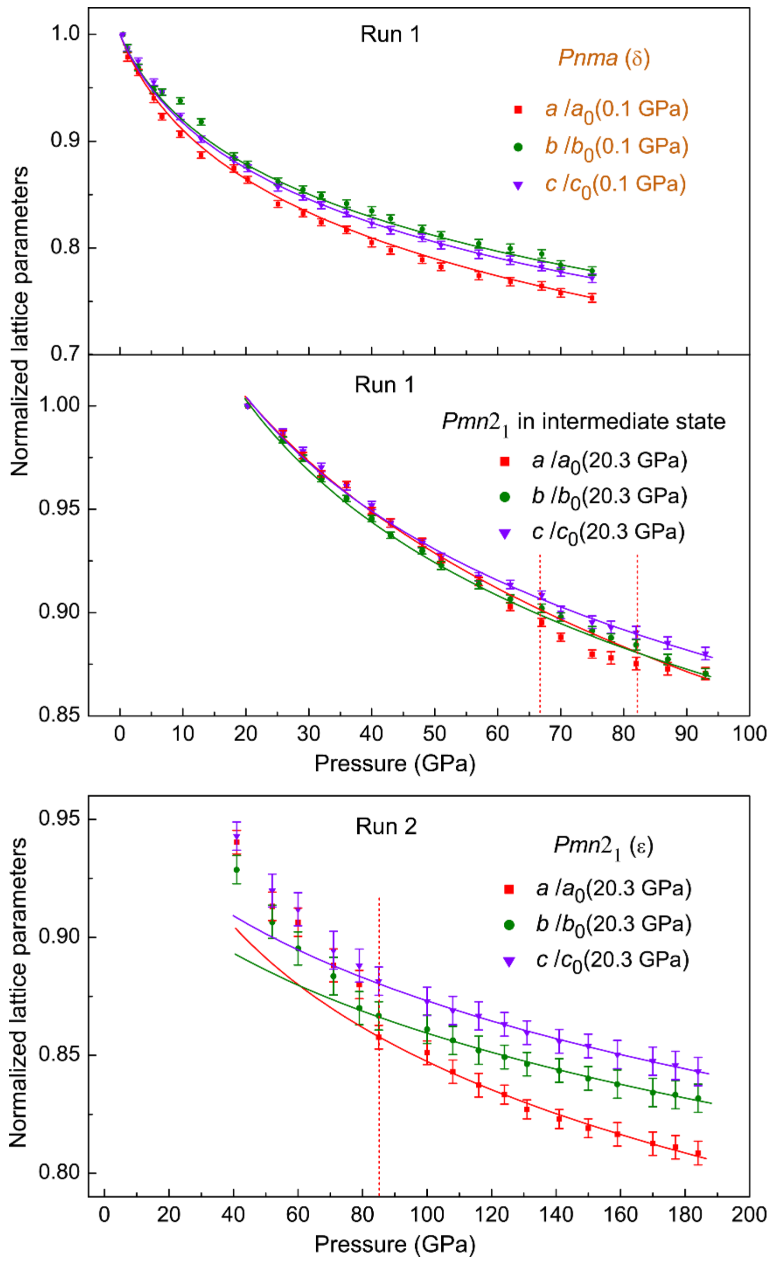

Supplementary Fig. 12. Lattice parameters of the *Pnma* (δ) and *Pmn*2_1 (ε) phases normalized to the values of the δ and ε phases at 0.1 GPa and 20.3 GPa, respectively. Top two panels, normalized lattice parameters obtained from the run 1 XRD. The solid lines are the fits of the *Pnma* phase (ambient – 75 GPa) and the *Pmn*2_1 phase in the intermediate state (20.3 – 82 GPa) using a modified third-order Birch-Murnaghan (B-M) equation of state where the unit cell volume is replaced with the cube of

individual lattice parameters[3]. The fits yield axial compressibility. The best fits for the *Pmn*2_1 phase in the intermediate state (20.3 – 82 GPa) are obtained by excluding the values within 62 – 82 GPa. The lattice constants of the *Pnma* phase deviate from the fit values between 6.7 – 12.9 GPa, concomitant with the beginning of the structural transition. The lattice constants, especially the *a* lattice constant, of the *Pmn*2_1 phase in the intermediate state show pronounced deviation from the fit values between 62 – 82 GPa (red dashed lines) that coincides with the insulator-semimetal-metal transition pressure. Bottom panel, normalized lattice parameters obtained from the run 2 XRD. The solid lines are the fits of the *Pmn*2_1 phase above 85 GPa where the structure stays in the high-pressure ε phase. The lattice parameters of the *Pmn*2_1 structure below 85 GPa deviate from the fitting lines significantly and have larger compressibility compared to those above 85 GPa, which is consistent with the compressibility change of the unit cell volume shown in Supplementary Fig. 13. This implies that the lattice parameters of the *Pmn*2_1 structure in the intermediate state is affected by the δ phase, and the system is not a simple mixing of the δ and ε phases, consistent with the run 1 XRD and simulation results. The error bars are based on the refinements and broadening of the XRD peaks at high pressures.

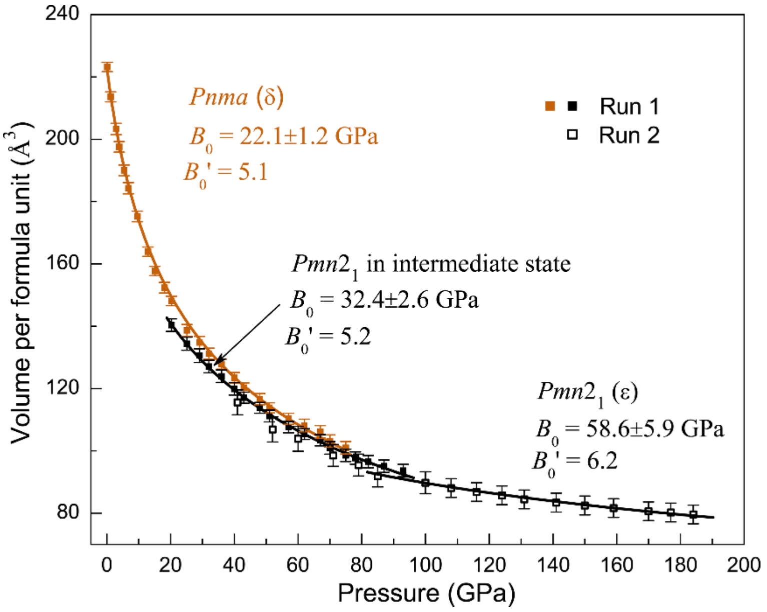

Supplementary Fig. 13. Pressure dependence of the volume per formula unit. Solid orange and black squares, and open black squares are the experimental data obtained from the run 1 and run 2 XRD, respectively. Fits to a third-order B-M equation of state are shown in orange and black solid lines for the *Pnma* and $Pmn2_1$ phase, respectively. Similar with the lattice parameters (Supplementary Fig. 12), the compressibility of the $Pmn2_1$ structure is different below versus above 85 GPa, although the volume change smoothly as a function of pressure. The pressure of 85 GPa in run 2 is consistent with the completion pressure of the δ-to-ε structural transition in run 1 (~82 GPa). The results show that the $Pmn2_1$ structure in the intermediate state (< 85 GPa) is more compressible than that in a pure ε phase (> 85 GPa). This implies that the compressibility of the $Pmn2_1$ structure in the intermediate state is affected by the δ phase, and the system is not a simple mixing of the δ and ε phases. The error bars come from the refinements and broadening of the XRD peaks that cause the uncertainties in the calculated lattice parameters.

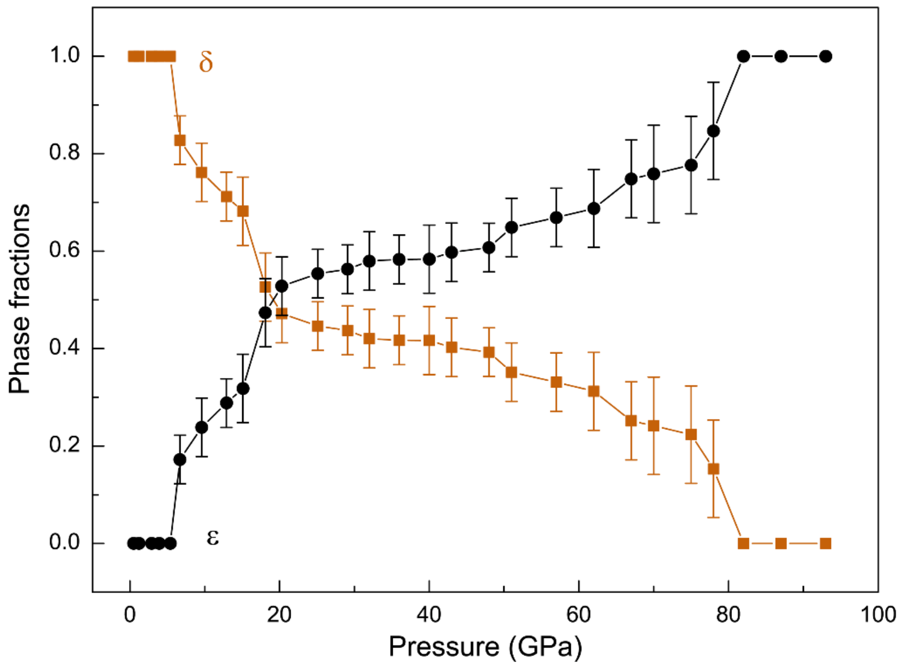

Supplementary Fig. 14. Estimated phase fractions as a function of pressure based on the Rietveld refinement results of run 1 XRD. The fraction of ε-$CsPbI_3$ increases with pressure at the expense of reducing δ-$CsPbI_3$. The error bars are from the refinements.

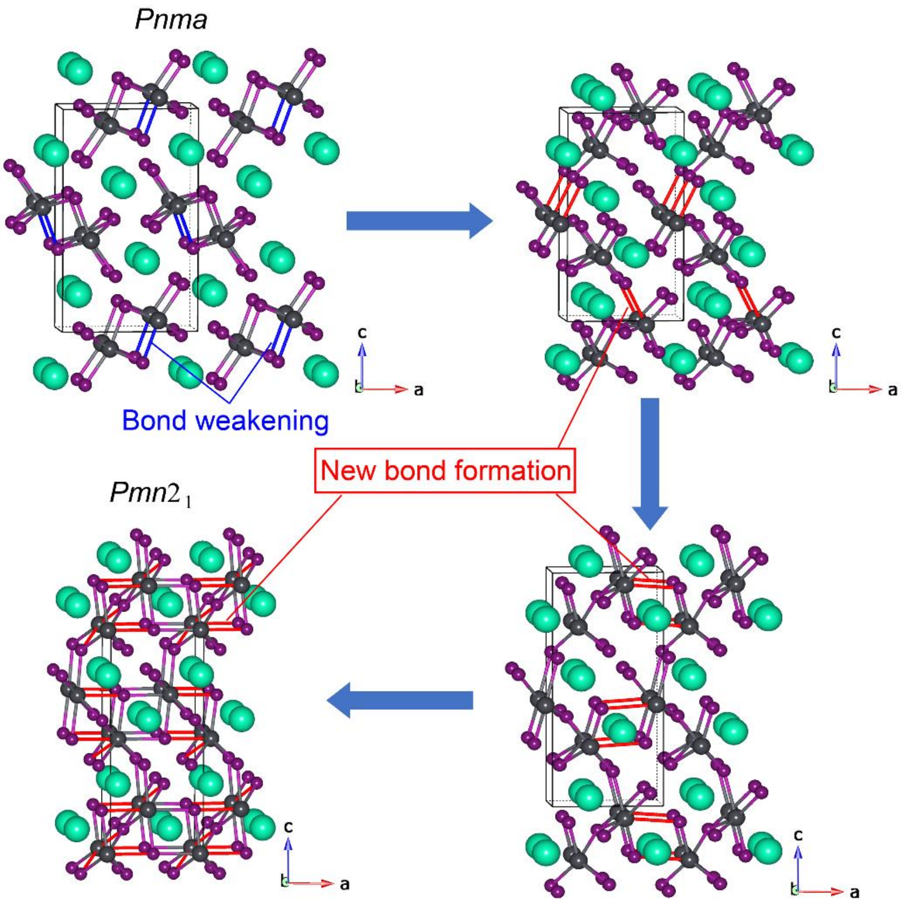

Supplementary Fig. 15. The predicted structural transition path from the initial *Pnma* (δ) to the high-pressure $Pmn2_1$ (ε) phase.

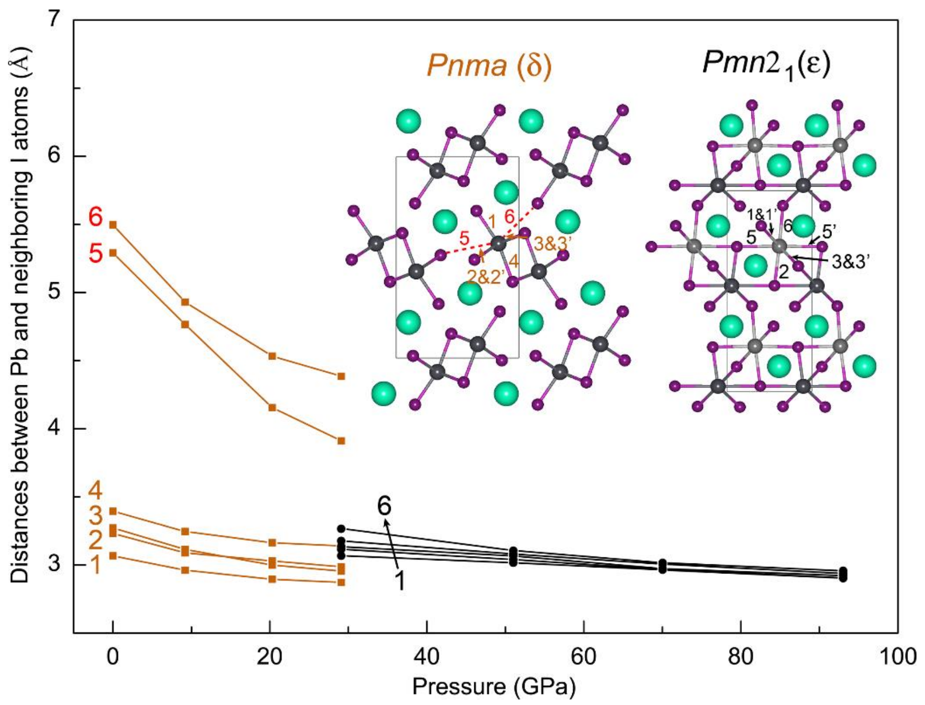

Supplementary Fig. 16. Distances between Pb and neighboring I atoms as a function of pressure. The results were obtained from the calculations by optimizing the atomic positions while fixing the lattice parameters to the experimental values at high pressures. For the *Pnma* (δ) phase, there are four unique Pb-I bonds within the PbI_6 octahedron that are labeled as bond 1-4 (orange squares). Bonds 2 & 2' and bonds 3 & 3' are pairs and have the same bond length. In the plot, we also included the distances of two pairs of non-bonded Pb and I atoms (5 and 6, red squares, dashed lines). As the structure evolves into the high-pressure ε phase, these two sets of atoms form bonds together, followed by the weakening and breakage of the initial Pb-I bond 4. Therefore, five different Pb-I bond lengths are present within the PbI_8 polyhedron of ε-$CsPbI_3$ (black circles, 1, 2, 3, 5 and 6). The 1 &1', 3 & 3', and 5 & 5' bonds are pairs and have the same bond length. All the Pb-I bonds shorten with pressure and converge to a similar bond length at 93 GPa. Likewise, the PbI_9 polyhedron of ε-

$CsPbI_3$ also has five unique Pb-I bonds and they change in a similar fashion with those of the PbI_8 polyhedron under pressure. The evolution of the Pb-I bond length with pressure, in particular the rapid approach of the initially non-bonded Pb and I (5 and 6), aligns well with the predicted transition path. Nevertheless, we must emphasize that the calculated evolution trajectory of some atoms would be different from the actual transition path. For example, according to the calculations, the bond 4 in the initial PbI_6 octahedron progressively shortens with pressure. However, this bond will elongate and weaken during the sluggish δ-to-ε phase transition until it is no longer a bond in the ε phase. The main reason for the discrepancy is that in lattice optimization, the symmetry is prohibited from changing, while the actual structural transition is a dynamic process that constantly involves symmetry breaking and phase fraction exchange as a function of pressure.

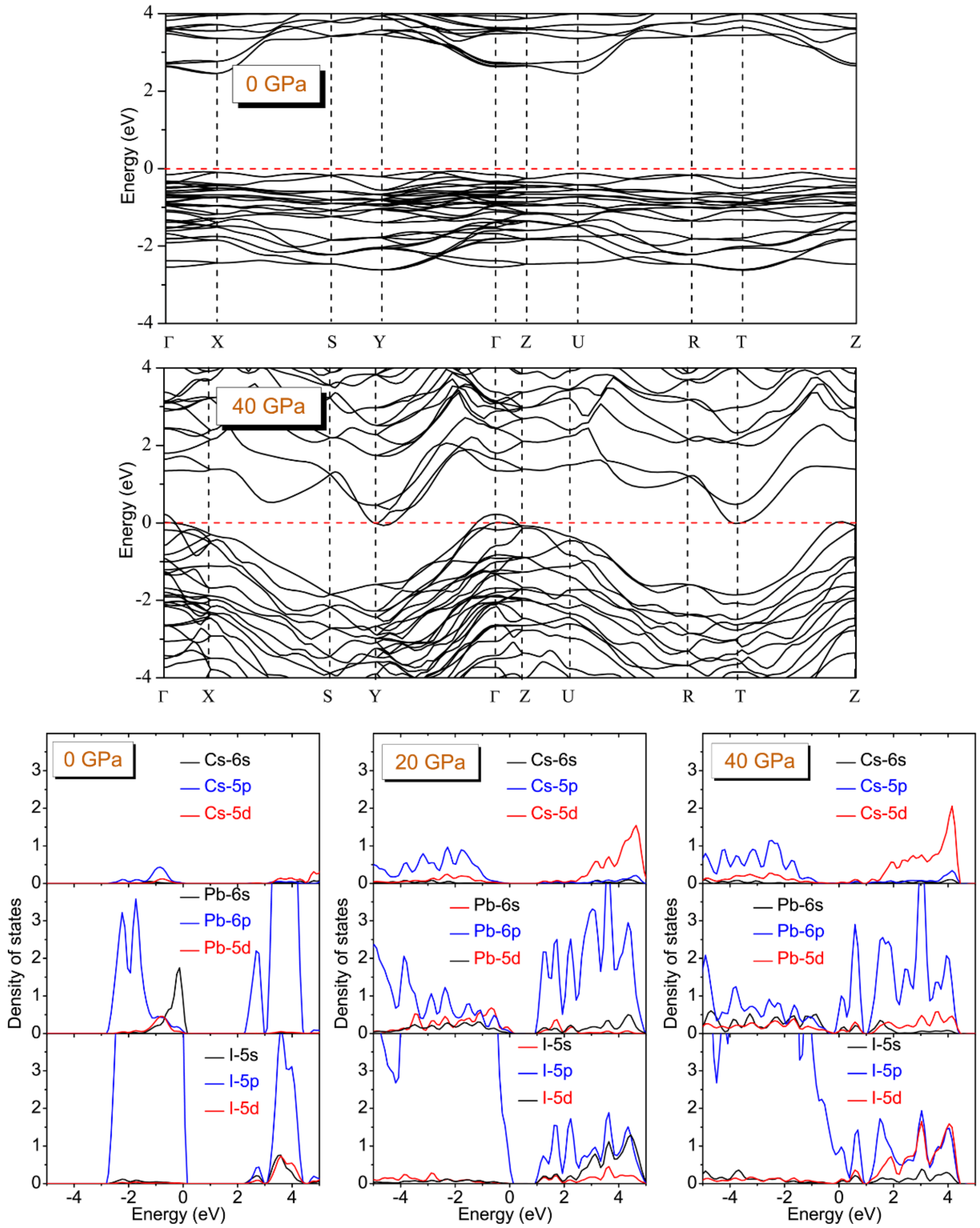

Supplementary Fig. 17. Electronic structure of the *Pnma* (δ) phase. Top two panels, calculated band structures at 0 and 40 GPa. Bottom nine panels, calculated density of states at 0, 20, and 40 GPa.

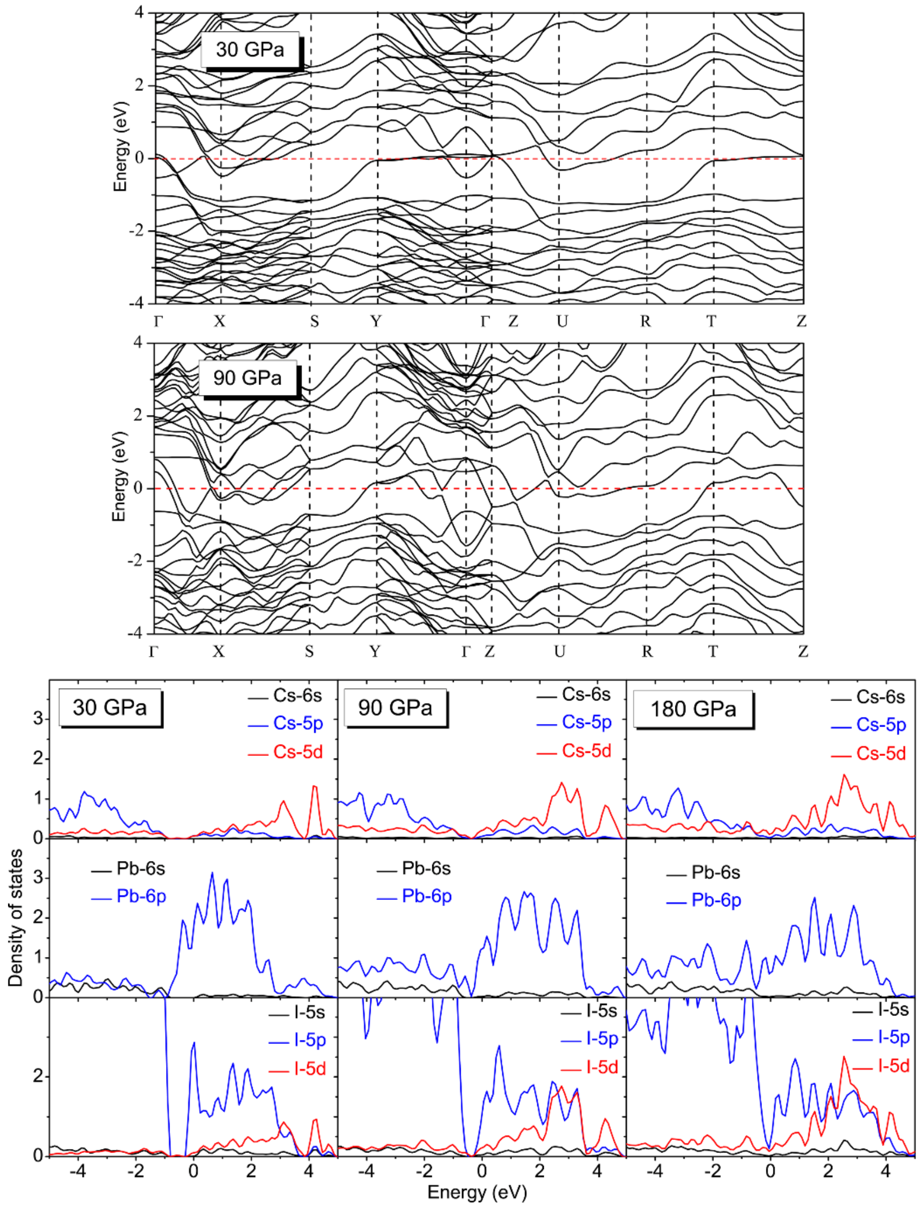

Supplementary Fig. 18. Electronic structure of the high-pressure metallic *Pmn*2_1 (ε) phase. Top two panels, calculated band structures at 30 and 90 GPa. Bottom nine panels, calculated density of states at 30, 90, and 180 GPa.

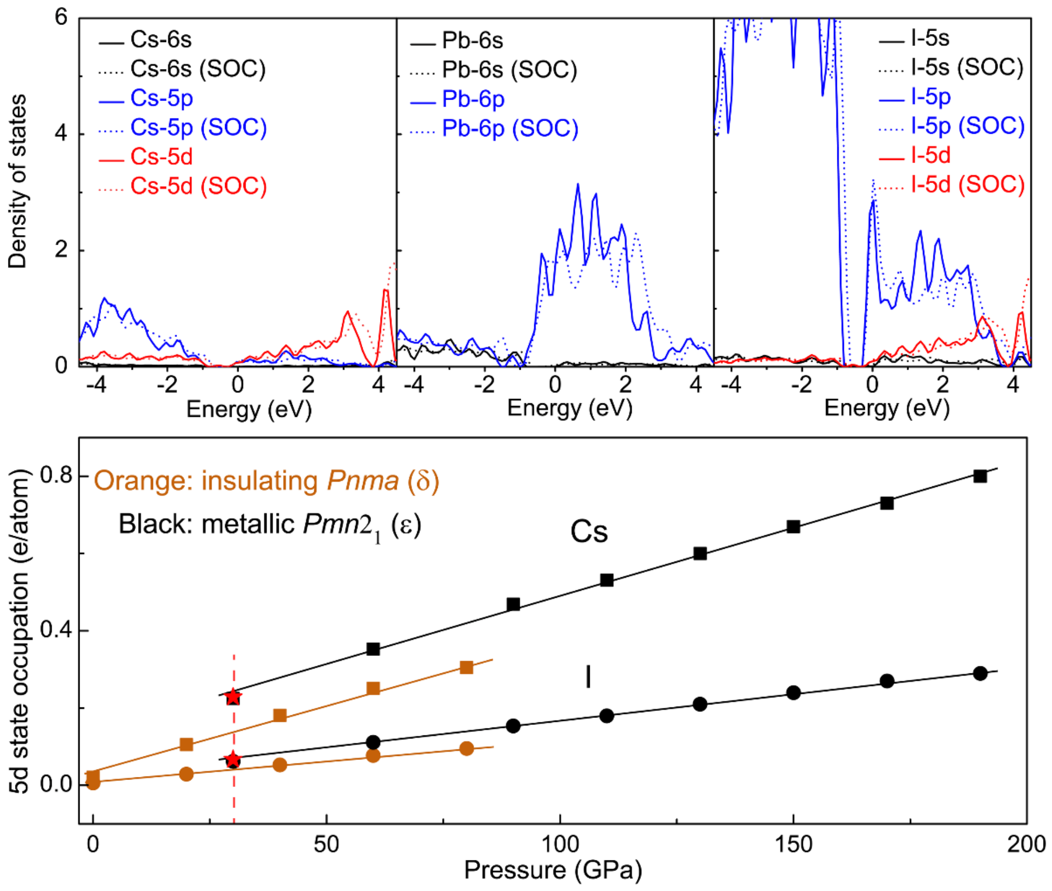

Supplementary Fig. 19. SOC effect on the electronic structure of the metallic *Pmn*2_1 (ε) phase. Top three panel, calculated density of states of the metallic *Pmn*2_1 (ε) phase at 30 GPa with (solid lines) and without (dashed lines) including the SOC effect. Bottom panel, calculated 5d state occupation of the Cs (squares) and I (circles) atoms in the starting δ (orange) and high-pressure ε (black) phases as a function of pressure without considering the SOC effect. The red stars represent the 5d state occupation of the Cs and I atoms in the ε phase at 30 GPa with considering the SOC effect. The red vertical dashed line indicates the transition pressure from the enthalpy calculations.

Supplementary Table 1. Structural details of the $Pmn2_1$ (ε) phase obtained from the experimental results at 82 GPa from run 1 and calculation results at 90 GPa.

Lattice parameters	Atomic positions					
a, *b*, *c* (Å)	Atom	Wyckoff	x	y	z	Uiso
Experiments	Cs	2a	0.6578	0.5	0.3129	0.179
(82 GPa)	Cs	2a	0.9843	0.0	0.6180	0.0765
Symmetry: $Pmn2_1$	Pb	2a	0.4008	0.0	0.1857	0.3924
a = 5.869±0.006	Pb	2a	0.9074	0.0	0.4376	0.2314
b = 4.516±0.005	I	2a	0.5739	0.5	0.1052	0.0893
c = 14.459±0.011	I	2a	0.6814	0.5	0.5048	0.0513
	I	2a	0.5840	0.5	0.9034	0.1365
	I	2a	0.8613	0.0	0.8065	0.2911
	I	2a	0.8236	0.0	0.0073	0.0633
	I	2a	0.8591	0.0	0.2089	0.4306
Simulations	Cs	2a	0.6376	0.5	0.3045	0.107
(90 GPa)	Cs	2a	0.9226	0.0	0.6063	0.107
Symmetry: $Pmn2_1$	Pb	2a	0.3594	0.0	0.2063	0.107
a = 5.830	Pb	2a	0.9206	0.0	0.4040	0.107
b = 4.423	I	2a	0.5739	0.5	0.1052	0.107
c = 14.512	I	2a	0.6814	0.5	0.5048	0.107
	I	2a	0.5840	0.5	0.9034	0.107
	I	2a	0.8613	0.0	0.8065	0.107
	I	2a	0.8236	0.0	0.0073	0.107
	I	2a	0.8591	0.0	0.2089	0.107

1 Gavriliuk, A. G., Mironovich, A. A. & Struzhkin, V. V. Miniature diamond anvil cell for broad range of high pressure measurements. *Rev. Sci. Instrum.* **80**, 043906 (2009).

2 Liang, Y. *et al.* New metallic ordered phase of perovskite $CsPbI_3$ under pressure. *Adv. Sci.* **6**, 1900399 (2019).

3 Runge, C. E. *et al.* Equation of state of $MgGeO_3$ perovskite to 65 GPa: comparison with the post-perovskite phase. *Phys. Chem. Miner.* **33**, 699-709 (2006).
