# Supplementary material for: Cesium-mediated electron redistribution and electron-electron interaction in high-pressure metallic CsPbI3
Source: Nat Commun. 2022 Nov 18;13:7067. doi: 10.1038/s41467-022-34786-5 (PMC9674642; doi:10.1038/s41467-022-34786-5)
Supplement: Supplementary file 3 — Editorial Assessment Report [file 41467_2022_34786_MOESM3_ESM.pdf]

nature portfolio

GUIDED OPEN ACCESS

nature physics | nature communications | communications physics

Contents of this report

1. **Manuscript details**: overview of your manuscript and the editorial team.
2. **Review synthesis**: summary of the reviewer reports provided by the editors.
3. **Editorial evaluations**: personalized evaluation and recommendation from all 3 journals.
4. **Annotated reviewer comments**: the referee reports with comments from the editors.
5. **Open research evaluation**: advice for adhering to best reproducibility practices.

About the editorial process

Because you selected the **Nature Portfolio Guided Open Access** option, your manuscript was assessed for suitability in three of our titles publishing high-quality work across the spectrum of physics research: ***Nature Physics***, ***Nature Communications***, **and** ***Communications Physics***. More information about Guided Open Access can be found here.

Collaborative editorial assessment

Your editorial team discussed the manuscript to determine its suitability for the Nature Portfolio Guided OA pilot. Our assessment of your manuscript takes into account several factors, including whether the work meets the **technical standard** of the Nature Portfolio and whether the findings are of **immediate significance** to the readership of at least one of the participating journals in the Nature Portfolio Guided Open Access physics cluster.

Peer review

Experts were asked to evaluate the following aspects of your manuscript:

- **Novelty** in comparison to prior publications;
- **Likely audience** of researchers in terms of broad fields of study and size;
- **Potential impact** of the study on the immediate or wider research field;
- **Evidence** for the claims and whether additional experiments or analyses could feasibly strengthen the evidence;
- **Methodological detail** and whether the manuscript is reproducible as written;
- Appropriateness of the **literature review**.

Editorial evaluation of reviews

Your editorial team discussed the potential suitability of your manuscript for each of the participating journals. They then discussed the revisions necessary in order for the work to be published, keeping each journal's specific editorial criteria in mind.

Journals in the Nature portfolio will support authors wishing to transfer their reviews and (where reviewers agree) the reviewers' identities to journals outside of Springer Nature.
If you have any questions about review portability, please contact our editorial office at guidedoa@nature.com.

nature portfolio

GUIDED OPEN ACCESS

nature physics | nature communications | communications physics

Manuscript details

Tracking number	Submission date	Decision date	Peer review type
GUIDEDOA-22-00395	Jan 18, 2022	May 20, 2022	Single-blind

Manuscript title

Cesium-involved electron transfer and electron-electron interaction in high-pressure metallic CsPbI3

Preprint: link if available

Author details

Yu Lin

Affiliation: Stanford Institute for Materials and Energy Sciences

Editorial assessment team

**Primary editor**	**Bart Verberck**<br>Home journal: ***Nature Physics & Nature Communications***<br>ORCID: **0000-0002-9601-8840**<br>Email: **bart.verberck@nature.com**
**Other editors consulted**	**Andrea Taroni**<br>Home journal: ***Nature Physics***<br>ORCID: **0000-0001-9550-5754**
**About your primary editor**	After 10 years in academia, Bart originally joined Nature Physics in 2013, where he handled topics from condensed-matter physics and plasma physics. He also launched a column called ‘Measure for Measure’ on aspects of metrology. In July 2017, Bart moved to Berlin to become Regional Executive Editor for the newly established Nature Portfolio office in Germany, which he helped develop as an editorial and publishing hub with a focus on local outreach. Since 2021, he combines that capacity with the role of Senior Consulting Editor at Nature Physics, where he

	covers soft-condensed-matter physics and has a consulting role for Nature Communications.

Editorial assessment and review synthesis

**Editor's summary and assessment**	This paper reports experiments and DFT calculations on CsPbI3, a halide perovskite. The authors find that in compressed CsPbI3, electron-electron interactions are important and that the system hosts a non-Fermi liquid state. They look at how the crystal structure and the electronic structure change when changing pressure, and reveal a transition from Fermi liquid-like behaviour to a non-Fermi liquid metal. Although the paper does not report findings of broad enough interest and relevance for the wider community of physicists that form the intended audience of Nature Physics, the study is definitely of high interest to a more specialist audience, and might be a good fit for Nature Communications, and very likely for Communications Physics.
**Editorial synthesis of reviewer reports**	The referees appreciate the structural and electronic characterization of the compound by means of experiments and computations over a wide range of pressures. They generally believe the work is sound. Some questions are raised, some of which are of a technical nature – these need to be addressed in a revised version of the manuscript. Further points raised by the referees can be used by the authors to enhance the relevance of their results.

Editorial evaluations

Nature Physics<br><br>Revision not invited	The reported results are unfortunately not of enough broad interest and relevance for the audience targeted by Nature Physics.
Nature Communications<br><br>Major revisions	For the manuscript to be considered further at Nature Communications, all technical issues raised by the referees must be satisfactorily addressed. The additional suggestions made by the referees should be taken into account to widen the interest of the paper.
Communications Physics<br><br>Minor revisions	For the manuscript to be considered further at Nature Communications, all technical issues raised by the referees must be satisfactorily addressed.

Next steps

**Editorial recommendation 1:**	Our top recommendation is to revise and resubmit your manuscript to *Nature Communications.* As mentioned above, using the suggestions made by the referees, the broader interest of the work needs to be convincingly demonstrated.
**Editorial recommendation 2:**	You may also choose to revise and resubmit your manuscript to *Communications Physics.* This option might be best if it is too difficult to take the non-technical issues raised by the referees into account.
**Note**	As stated on the previous page *Journal C* is not inviting a revision at this time. Please keep in mind that the journal will not be able to consider any appeals of their decision through Guided Open Access.

Revision

To follow our recommendation, please upload the revised manuscript files using **the link provided in the decision letter**. Should you need assistance with our manuscript tracking system, please contact Adam Lipkin, our Nature Portfolio Guided OA support specialist, at guidedOA@nature.com.

Revision checklist

- ☐ Cover letter, stating to which journal you are submitting
- ☐ Revised manuscript
- ☐ Point-by-point response to reviews
- ☐ Updated Reporting Summary and Editorial Policy Checklist
- ☐ Supplementary materials (if applicable)

Submission elsewhere

If you choose not to follow our recommendations, you can still take the reviewer reports with you.

Option 1: Transfer to another Nature Portfolio journal
Springer Nature provides authors with the ability to transfer a manuscript within the Nature Portfolio, without the author having to upload the manuscript data again. To use this service, **please follow the transfer link provided in the decision letter.** If no link was provided, please contact guidedOA@nature.com.

> *Note that any decision to opt in to In Review at the original journal is not sent to the receiving journal on transfer. You can opt in to In Review at receiving journals that support this service by choosing to modify your manuscript on transfer.*

Option 2: Portable Peer Review option for submission to a journal outside of Nature Portfolio
If you choose to submit your revised manuscript to a journal at another publisher, we can share the reviews with another journal outside of the Nature Portfolio if requested. You will need to request that the receiving journal office contacts us at guidedOA@nature.com. We have included editorial guidance below in the reviewer reports and open research evaluation to aid in revising the manuscript for publication elsewhere.

Annotated reviewer reports

The editors have included some additional comments on specific points raised by the reviewers below, to clarify requirements for publication in the recommended journal(s). However, please note that all points should be addressed in a revision, even if an editor has not specifically commented on them.

Reviewer #1 information

**Expertise**	X-ray diffraction, imaging and spectroscopy, materials under high-pressure
**Editor's comments**	This reviewer appreciates the importance of the work reported, specifically the discovery of the transition form non Fermi-liquid (FL) like to FL like. A few technical points need to be resolved, though, and some suggestions can be considered.

Reviewer #1 comments

Section	Annotated Reviewer Comments
**Remarks to the Author: Overall significance**	Ke et al. reported the low-temperature electrical transport study on CsPbI3 under high pressure up to 183 GPa and discussed the Fermi liquid (FL) - like behavior. In the low temperature region, especially below the Debye-temperature, the R(T) vs T^n dependence were analyzed, which yields the carrier transportation type and concentration. They reported a sluggish structure phase transition from Pnma to Pmn21, while the deviation from a FL-like state gradually disappears after entering metallic phase at 80 GPa to highest pressure 186 GPa. The A-site cation (Cs) is found to play a prominent role in the electronic structure at high pressure rather than indirect effect at ambient pressure. Overall, this work is well written and address the important issue on the low-temperature transport study in the electron-electron interaction behavior of metallic phase CsPbI3 at high pressure. I would recommend the paper is acceptable for publication after addressing several concerns I have.
**Remarks to the Author: Impact**	The discovery of non FL-like to FL-like behavior is the major contribution to the community in the metallic phase, which may generate a wave of research to explore similar behavior in other systems.
**Remarks to the Author: Strength of the claims**	Concerns need to be addressed in the revision.<br>1) The important discovery is the n index in the R(T) curve fitting at pressure 80 to 186 GPa follows a curve gradually approaching 2, the ideal FL-like state. The discussion on the origin of n deviated away from 2 is not quite clear. The XRD study shows a sluggish transition from the pnma （δ phase） to Pmn21 （ε phase）, and completed around 82 GPa, similar pressure range as the RT resistivity behavior. The XRD data provide good evidence of δ phase pressure range, but very limit for the ε phase structural information (up to 93 GPa) for the non FL-like to FL-like electronic behavior evolution. So the structural information at higher pressure XRD data would

	be very useful .<br>2) The claim of insulator to metal conductivity transition at 80 Gpa is questionable. The R(T) between 50 GPa and 80 Gpa does not show a typical insulating behavior, but rather a semi-metal. The Band structure and DOS calculation also show a significant change above 30 GPa. Please revise your claim accordingly.<br><br>**Please address these points in a revised version of your manuscript.**<br><br>3) This work provides a good example for the electronic property dominated by Pb-I frame work at low pressure, but the cation (Cs this case) provides direct and pronounced contribution at high pressure. Since there are numerical publications on many inorganic and organic-inorganic hybrid perovskites with similar wide band gap semiconducting to metallic phase transition, can authors extend their discovery to other systems? If so, is the non FL-like to FL-like electronic behavior can be considered as a general mechanism or some limitation.<br><br>**Please carefully consider this point; in order to be suitable for publication Nature Communications, broad enough relevance is required.**<br><br>4) It would be useful to see if the structure and electronic transitions are reversible upon decompression. If the high pressure phase can be reserved to ambient condition, the FL-like behavior would be useful for ambient pressure application; if the structure is reversed back to pristine phase, it may be useful to run the compression experiment again to see any improvement in conductivity can be obtained since the contact between nano-grains would be better.<br><br>**Including a discussion on this point would add value to your manuscript.**
**Remarks to the Author: Reproducibility**	As mentioned in concern 4 above, it would be useful to conduct a decompression and second round compression experiment on this system to clear some uncertainty.

Reviewer #2 information

**Expertise**	Light emitting and energy storage materials and applications
**Editor's comments**	This reviewer appreciates the study, recognizes the potential relevance for tuning electron interactions in halide perovskites. He/she raises some points and questions, the addressing of which would enhance the appeal of the work further.

Reviewer #2 comments

Section	Annotated Reviewer Comments
**Remarks to the Author: Overall significance**	In this manuscript, to illustrate the electronic structures and carrier scattering mechanisms in metal halide perovskites at low temperature combined with high pressure conditions, the authors studied electronic states in CsPbI3 over a vast pressure-temperature space of 0.1-186 GPa and 2-300 K. The experimental results show that by compressing CsPbI3 to 80 GPa, the insulating phase transforms to a metallic state, and a Fermi liquid-like state is observed with further compression to 186 GPa. Through the first-principles DFT calculations, the authors unravel that the Cs atom has a direct and pronounced contribution to the electrical properties of CsPbI3 at high pressure.
**Remarks to the Author: Impact**	This work presents an interesting strategy for tuning the electronic interaction in halide perovskites for realizing intriguing electronic states.
**Remarks to the Author: Strength of the claims**	The following comments are provided to strength the claims of this work.<br><br>1) The authors mentioned that the δ-to-ε structural evolution involves a sequence of Pb-I bond breaking in the starting PbI6 octahedral chains and the formation of Pb-I bonds between adjacent chains under the compression. To fully illustrate this interesting structural evolution, please provide the bond lengths and bond energies of Pb-I bonds under different structures. How does the high pressure induce the bond disconnection/connection and how about the energy transformation during this process?<br>2) It is very interesting to see the sample color change with phase transition process. Is the entire phase transition process reversible? Will the color of the sample change from black to red and then to yellow by releasing the compressive force?<br>3) On page 13, the authors believed that "pressure-induced defects and disorder are not the root cause of the deviation in the high-pressure metallic phase because they are present throughout the compression process". How do the authors know the defects and disorder exist throughout the testing process? Does the type of defect not change during the compression of the sample? If the type of defect changes, will there be an impact on the electrical transport of the sample?<br><br>**Please carefully consider the above remarks. It is likely that with the suggested additional clarifications, your manuscript can become suitable for publication in Nature Communications.**
**Remarks to the Author: Reproducibility**	The reviewer noted that the starting sample for this study is the CsPbI3 powder. The crystal orientation in the powder sample is random, which means that specific direction of the pressure applied on the CsPbI3 can not be

	determined. Dose this uncertainty influence the repeatability of testing results? Is it possible to employ the single CsPbI3 crystal to carry out the experiments?<br><br>**Please clarify this point as it is indeed relevant to the issue of reproducibility.**

Open research evaluation

Data availability
**Data availability statement**
Thank you for including a Data Availability statement. However, we do not see any raw-data sets. Can you please look into this. Also, the data availability statement must make the conditions of access to the "minimum dataset" that are necessary to interpret, verify and extend the research in the article, transparent to readers.<br><br>See here for more information about formatting your Data Availability Statement.
**Code availability statement**
For all studies using custom code or mathematical algorithm that is deemed central to the conclusions, a statement must also be included under the heading "Code availability", indicating whether and how the code or algorithm can be accessed, including any restrictions to access. Code availability statements should be provided as a separate section after the data availability statement but before the References.<br><br>You mention codes for reproducing the data plots, but at the indicated repository (https://repository.kaust.edu.sa/handle/10754/671206), we do not see any code. Can you please look into this, and update your Code Availability Statement accordingly.
**Other data requests**
Springer Nature strongly supports data sharing and believes that all datasets on which the conclusions of the paper rely should be available to readers. We encourage authors to ensure that their datasets are either deposited in publicly available repositories (where available and appropriate) or presented in the main manuscript or additional supporting files whenever possible.<br><br>Please see Springer Nature's information on recommended repositories here.

Data availability
All source data underlying the graphs and charts presented in the main figures must be made available as Supplementary Data (in Excel or text format) or via a generalist repository (eg, Figshare or Dryad). This is mandatory for publication in a Nature Portfolio journal, but is also best practice for publication in any venue. In the present paper, Figure 1 requires associated source data.
**Reporting & reproducibility**
Nature Portfolio journals allow unlimited space for Methods. The Methods must contain sufficient detail such that the work could be repeated. It is preferable that all key methods be included in the main manuscript, rather than in the Supplementary Information. Please avoid use of “as described previously” or similar, and instead detail the specific methods used with appropriate attribution.
**Statistics and data presentation**
The meaning of all error bars/bands and how they were calculated should be described within the captions of all figures in which they occur. If they represent standard deviations (or absolute minima and maxima) then this can be simply stated as such, but if not, more detail is required.
